# Analytical comparisons of SARS-COV-2 detection by qRT-PCR and ddPCR with multiple primer/probe sets

**DOI:** 10.1080/22221751.2020.1772679

**Published:** 2020-06-04

**Authors:** Xinjin Liu, Jiangpeng Feng, Qiuhan Zhang, Dong Guo, Lu Zhang, Tao Suo, Wenjia Hu, Ming Guo, Xin Wang, Zhixiang Huang, Yong Xiong, Guozhong Chen, Yu Chen, Ke Lan

**Affiliations:** a State Key Laboratory of Virology, Modern Virology Research Center, College of Life Sciences, Wuhan University, Wuhan, People’s Republic of China; b State Key Laboratory of Virology, Renmin Hospital, Wuhan University, Wuhan, People’s Republic of China; c Department of Infectious Disease, Zhongnan Hospital, Wuhan University, Wuhan, People’s Republic of China; d Frontier Science Center for Immunology and Metabolism, Wuhan University, Wuhan, People’s Republic of China

**Keywords:** SARS-CoV-2, diagnosis, digital PCR, real time PCR, false positive, false negative

## Abstract

Different primers/probes sets have been developed all over the world for the nucleic acid detection of SARS-CoV-2 by quantitative real time polymerase chain reaction (qRT-PCR) as a standard method. In our recent study, we explored the feasibility of droplet digital PCR (ddPCR) for clinical SARS-CoV-2 nucleic acid detection compared with qRT-PCR using the same primer/probe sets issued by Chinese Center for Disease Control and Prevention (CDC) targeting viral ORF1ab or N gene, which showed that ddPCR could largely minimize the false negatives reports resulted by qRT-PCR [Suo T, Liu X, Feng J, et al. ddPCR: a more sensitive and accurate tool for SARS-CoV-2 detection in low viral load specimens. medRxiv [Internet]. 2020;2020.02.29.20029439. Available from: https://medrxiv.org/content/early/2020/03/06/2020.02.29.20029439.abstract]. Here, we further stringently compared the performance of qRT-PCR and ddPCR for 8 primer/probe sets with the same clinical samples and conditions. Results showed that none of 8 primer/probe sets used in qRT-PCR could significantly distinguish true negatives and positives with low viral load (10^−4^ dilution). Moreover, false positive reports of qRT-PCR with UCDC-N1, N2 and CCDC-N primers/probes sets were observed. In contrast, ddPCR showed significantly better performance in general for low viral load samples compared to qRT-PCR. Remarkably, the background readouts of ddPCR are relatively lower, which could efficiently reduce the production of false positive reports.

The pandemic of severe acute respiratory syndrome coronavirus (SARS-CoV-2, also refers as HCOV-19) [1,2] has raised an urgent requirement for clinical pathogen diagnosis. The gold standard method, quantitative real time polymerase chain reaction (qRT-PCR) assay, is being widely used for rapid detection of the SARS-CoV-2 infection. However, the problem of qRT-PCR with inaccurate results was increasingly exposed. Our previous study showed that significant numbers of false negative reports (FNRs) of qRT-PCR are inevitable, which may compromise the timely diagnosis, early treatment, prevention of transmission, and assessment of discharge criteria [3]. The complementary use of droplet digital PCR (ddPCR) for SARS-CoV-2 nucleic acid detection could largely minimize the FNRs resulted by qRT-PCR with the same primer/probe sets issued by Chinese Center for Disease Control and Prevention (CCDC) [3]. Of note, an increasing number of false positives reports (FPRs) of qRT-PCR were observed in the practices of SARS-CoV-2 diagnosis for convalescent patients and asymptomatic infected patients recently. Moreover, it has been reported that the qRT-PCR performance of each primer-probe set is different from others, and many primer/probe sets have background amplification with SARS-CoV-2 negative nasopharyngeal swabs [4], leading to the inconclusive results. In the present study, by using the cDNAs of nasopharyngeal swabs from healthy people and SARS-CoV-2 infection patients, we stringently compared the performance of qRT-PCR and ddPCR for 8 commonly used primer/probe sets with the same conditions.

Nasopharyngeal swabs of healthy people (IgM/IgG negative) and patients with SARS-CoV-2 infection were collected from Renmin Hospital of Wuhan University. Total RNAs were extracted and reversely transcribed to cDNAs, which were pooled together then, respectively. Serial 10-fold dilutions of pooled cDNAs from healthy people or patients were conducted and subjected to qRT-PCR or ddPCR assays simultaneously using the primer/probe sets issued by different institutions (Table S1), including CCDC [5], Hong Kong University (HKU) [6], Charité (Universitätsmedizin Berlin Institute of Virology, Germany) [7], and United States Center for Disease Control and Prevention (UCDC) [8]. All the procedures of qRT-PCR and ddPCR had been described before, following the basic instructions of the organizations that designed these primer/probe sets and the instrument manufacturer in general. (see Methods in supplementary materials) [3]. Nasopharyngeal swabs of healthy people were used as mock control. The maximum value of mock control for each primer/probe set detected by qRT-PCR or ddPCR was used as cut-off threshold, respectively, to distinguish negative and positive. However, the criterion such as the limit of detection (LoD) for ddPCR or cut-off cycle threshold (CT) value for qRT-PCR were usually determined using universal methods with standard materials or lots of patients’ samples, which were not included in this study [9,10].

As shown in Figure 1A, in the mock control using the samples from healthy people, CT values <40 for the UCDC-N1 (10/10), UCDC-N2 (6/10), CCDC-N (5/10) sets were detected, indicating amplification of nonspecific products. Moreover, the background CT values of mock samples overlapped with that of the 10^−4^ and 10^−3^ diluted patient samples, which suggests that the abilities of UCDC-N1, UCDC-N2 and CCDC-N sets to differentiate between true positives and negatives at low virus concentration are limited, leading to FPRs. In contrast, among the primer/probe sets without background CT values in the mock control, the CT values of UCDC-N3, HKU-N, Charité-E and HKU-ORF sets at dilution of 10^−4^ were around 40 (not detected region), which could not identify the positive samples entirely, leading to FNRs. The most sensitive primer/probe set for qRT-PCR might be CCDC-ORF, which could detected 3/10 (30%) of positive samples at dilution of 10^−4^ with CT values <38, indicating positive according to the criteria of CCDC [5]. However, the CT values of CCDC-ORF for qRT-PCR ranged from 35.5 to 39.4, showing low consistency. The repeatability and consistency are greatly improved at dilution of 10^−2^ and 10^−1^ for all primer/probe sets, which demonstrated that qRT-PCR is still a reliable method for the detection of normal viral load samples. Of note, based on the accuracy with 95% detection ratio in qRT-PCR assay, UCDC-N1, UCDC-N3, HKU-N, CCDC-N and HKU-ORF could be used to define the viral load when their CT values are <34 (Table S2).Figure 1.Results of qRT-PCR and ddPCR for different primers/probes sets. (A) Results of qRT-PCR with different primer/probe sets. Dilution multiples (converted to log_10_) were plotted on the X axis versus measured Ct values of qRT-PCR on the Y axis. CT value ≥40 were plotted as not detected (ND). (B) Results of ddPCR with different primer/probe sets. Dilution multiples (converted to log_10_) were plotted on the X axis versus measured values of ddPCR (converted to log_10_) on the Y axis. Value with 0 copies/ reaction were plotted as ND. For each primer-probe set, we show the range of measured cycle threshold and concentration values obtained with mock samples from healthy people (IgM/IgG negative) in green-shaded areas. Values of patients’ samples beyond the maximum values of mock samples were judged as positive.

**Figure:**
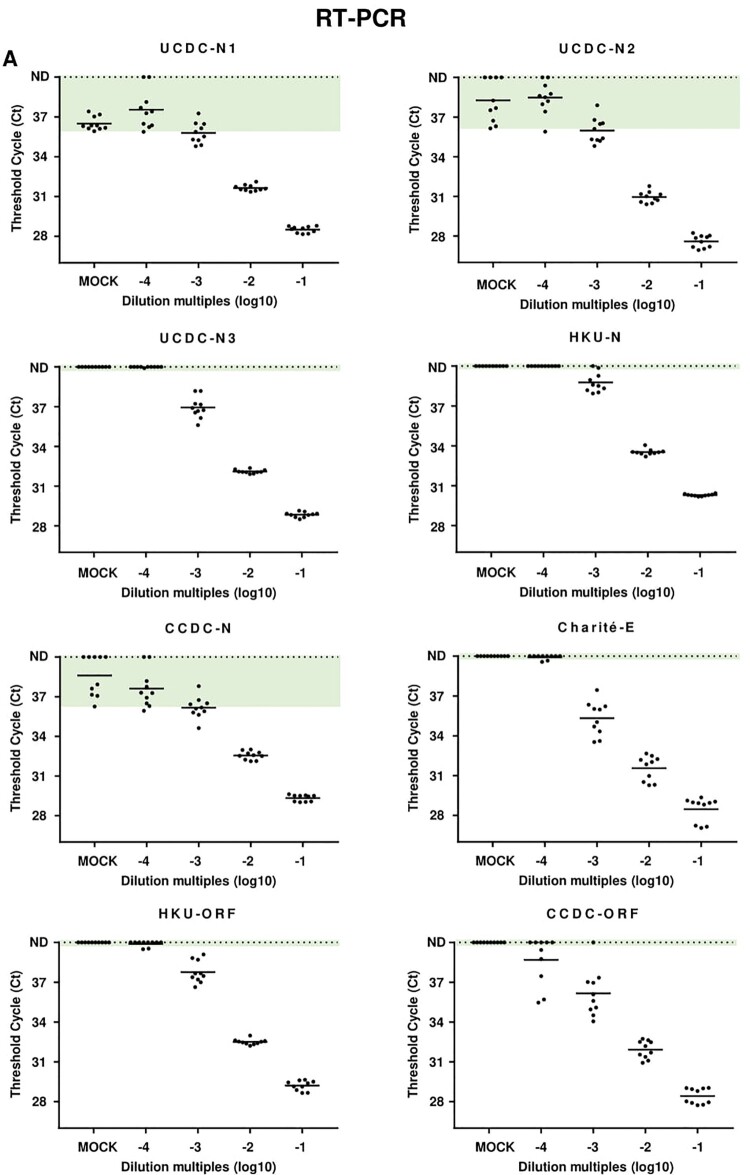

**Figure:**
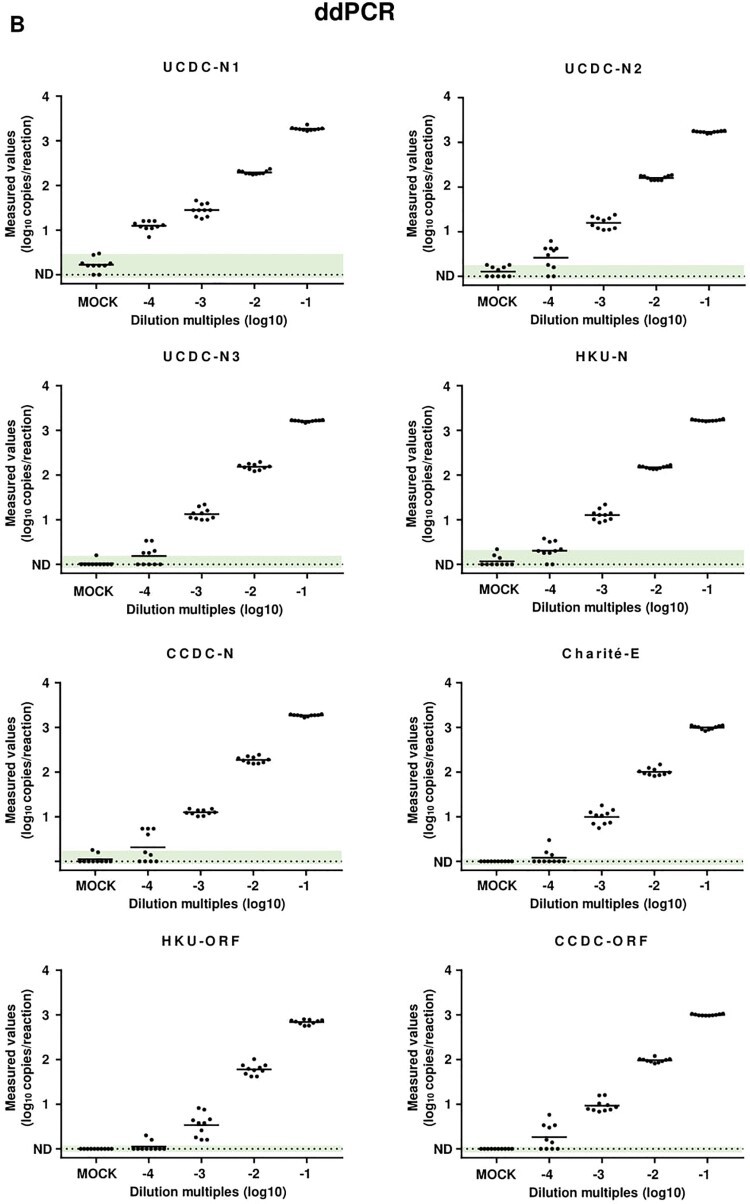

Accordingly, the same samples were detected by ddPCR using the same primer/probe sets, which showed that the performances of ddPCR for the detection of low viral load samples were significantly improved in general (Figure 1B). In the mock control of ddPCR, although UCDC-N1 and N2 showed high percentages of background signals with 8/10 and 5/10, respectively, they could still significantly distinguish the low viral load samples (dilution of 10^−4^) from mock by generating relatively higher signals. Moreover, UCDC-N3, HKU-N and CCDC-N showed low percentages of background signals with 1/10, 3/10 and 2/10, respectively. Meanwhile, Charité-E, HKU-ORF and CCDC-ORF did not show any background signal in ddPCR assays for mock control. Remarkably, all low and no background primer/probe sets could produce correct positive reports to varying degrees ranging from 2/10 (HKU-ORF) to 6/10 (CCDC-ORF), according to the maximum value of each primer/probe set in the mock control. Of note, the ddPCR readouts of both CCDC-N and CCDC-ORF for mock samples were below the limitation of detection (LoD) determined in our previous study [3]. Therefore, ddPCR could significantly reduce both the FNRs and FPRs in the detection of low SARS-CoV-2 load samples, when the negative threshold defined as <1.0–3.0 copies/reaction based on the performance of each primer/probe set (Table S3).

Our results showed that the ddPCR method significantly reduces the inaccurate results including FNRs or FPRs in the low viral load samples compared to qRT-PCR. Furthermore, 8 primer/probe sets showed different characterizations or limitations in the applications of qRT-PCR or ddPCR for SARS-CoV-2 detection, which could help for the selection of appropriate methods as well as primer/probe sets. Remarkably, due to the different performances of each primer/probe set in ddPCR, the optimization such as the design or selection of proper primer/probe set and the corresponding annealing temperature, LoD for the negative threshold determination could help to eliminate false positives or negatives when used for clinical diagnosis of viral infection. Meanwhile, due to the possible FPRs or FNRs resulted from current standard qRT-PCR detection of SARS-CoV-2, the better choice of clinical practice would be a comprehensive approach including nucleic acid test, imaging, serum test, as well as the judgement by experienced clinical doctors instead of solely depending on qRT-PCR.

Nevertheless, ddPCR also present some disadvantages. Although ddPCR is independent of a traditional standard curve, precisely and accurately defined calibrant materials or gold standards are still required to ensure commutability between molecular diagnostics laboratories. In addition, a ddPCR assay for a 96-well plate would take approximately two times longer than our current clinical qRT-PCR assay even with automation equipment. Finally, ddPCR is currently more expensive than qPCR per test with specialized instrumentation and consumables. In conclusion, qRT-PCR is suitable for large scale diagnosis of viral infection in normal viral load samples. However, ddPCR would be more ideal method for quantitation with special requirements for sensitivity and precision [11].

## Supplementary Material

EMI-SM_0502.docx
