# Supplementary material for: Analytical comparisons of SARS-COV-2 detection by qRT-PCR and ddPCR with multiple primer/probe sets
Source: Emerg Microbes Infect. 2020 Jun 4;9(1):1175–9. doi: 10.1080/22221751.2020.1772679 (PMC7448863; doi:10.1080/22221751.2020.1772679)
Supplement: EMI-SM_0502.docx [file TEMI_A_1772679_SM9137.docx]

**Supplementary Materials for**

**Analytical comparisons of SARS-COV-2 detection by RT-PCR and ddPCR with different primer/probe sets**

**Materials and methods**

***Specimen collection and RNA extraction***

Nasopharyngeal swabs were soaked in 500 μl PBS and vortexed with a diameter of 3 mm beads (Novastar, China) for 15 seconds immediately. Total RNA were extracted from the supernatant using the QIAamp viral RNA mini kit (Qiagen) following the manufacturer’s instruction. cDNA was synthesized using PrimeScript RT Master Mix (TakaRa) with random primer and oligo dT primer. Subsequent tests of both RT-PCR and ddPCR were conducted using the primers/probes from different institutes (Table S1) with 10 replicates for each dilution. To avoid the risk of viral infection and false positive results potentially due to the laboratory contamination, all the experiments were done inside the biosafety cabinet in negative pressure biosafety laboratory using filter tips.

***Droplet Digital PCR workflow***

All the procedures follow the manufacture instructions of the QX200 Droplet Digital PCR System using supermix for probe (no dUTP) (Bio-Rad) with primers/probes mix (final concentrations of 900 nM and 250 nM, respectively) and 2μl template(cDNA) in a final volume of 20 μl. Cycling protocol was set at 95°C for 10 min, followed by 40 cycles of 94°C for 30 s and 60°C for 1 min followed by an infinite 4-degree hold. More details were described as before [1].

***RT-PCR***

The procedures of RT-PCR follow the manufacturer’s instructions (Shanghai BioGerm Medical Biotechnology). A 25 μl reaction mix was set up containing 2 μl of template (cDNA), 12 μl of reaction buffer, 4 μl of primers and probe mix, 4 µl of enzyme mix and 2 μl of ddH2O. Thermal cycling was performed at 95°C for 5 min and then 40 cycles of 95°C for 10s, 55°C for 40 s in BIO-RAD CFX96 Touch Real-Time PCR Detection system (Bio-Rad).

***Data analysis***

Analysis of the ddPCR data was performed with Quanta Soft analysis software v.1.7.4.0917 (Bio-Rad) to calculate the concentration of the target. The positive populations for each primer/probe are identified using positive and negative controls with single (i.e., not multiplexed) primer–probe sets. In addition, plots of results of ddPCR and RT-PCR for different primers were conducted with GraphPad Prism 7.00.

**Table S1. Primers and probes from** **different institutes for SARS-CoV-2 diagnostics.**

| Institute | Primer/Probe | Sequence (5’-3’) | Reference |
| --- | --- | --- | --- |
| CCDC | CCDC-ORF-Fwd | CCCTGTGGGTTTTACACTTAA | [2] |
|  | CCDC-ORF-Rev | ACGATTGTGCATCAGCTGA |  |
|  | CCDC-ORF-Probe | CCGTCTGCGGTATGTGGAAAGGTTATGG |  |
|  | CCDC-N-Fwd | GGGGAACTTCTCCTGCTAGAAT |  |
|  | CCDC-N-Rev | CAGACATTTTGCTCTCAAGCTG |  |
|  | CCDC-N-Probe | TTGCTGCTGCTTGACAGATT |  |
| HKU | HKU-ORF-Fwd | TGGGGYTTTACRGGTAACCT | [3] |
|  | HKU-ORF-Rev | AACRCGCTTAACAAAGCACTC |  |
|  | HKU-ORF-Probe | TAGTTGTGATGCWATCATGACTAG |  |
|  | HKU-N-Fwd | TAATCAGACAAGGAACTGATTA |  |
|  | HKU-N-Rev | CGAAGGTGTGACTTCCATG |  |
|  | HKU-N-Probe | GCAAATTGTGCAATTTGCGG |  |
| Charité | Charité_F | ACAGGTACGTTAATAGTTAATAGCGT | [4] |
|  | Charité_R | ATATTGCAGCAGTACGCACACA |  |
|  | Charité_P1 | ACACTAGCCATCCTTACTGCGCTTCG |  |
| UCDC | UCDC_N1-F | GAC CCC AAA ATC AGC GAA AT | [5] |
|  | UCDC_N1-R | TCT GGT TAC TGC CAG TTG AAT CTG |  |
|  | UCDC_N1-P | ACC CCG CAT TAC GTT TGG TGG ACC |  |
|  | UCDC_N2-F | TTA CAA ACA TTG GCC GCA AA |  |
|  | UCDC_N2-R | GCG CGA CAT TCC GAA GAA |  |
|  | UCDC_N2-P | ACA ATT TGC CCC CAG CGC TTC AG |  |
|  | UCDC_N3-F | GGG AGC CTT GAA TAC ACC AAA A |  |
|  | UCDC_N3-R | TGT AGC ACG ATT GCA GCA TTG |  |
|  | UCDC_N3-P | AYC ACA TTG GCA CCC GCA ATC CTG |  |

| UCDC-N1 | | | | | UCDC-N2 | | | | |
| --- | --- | --- | --- | --- | --- | --- | --- | --- | --- |
| MOCK | 10^-4^ | 10^-3^ | 10^-2^ | 10^-1^ | MOCK | 10^-4^ | 10^-3^ | 10^-2^ | 10^-1^ |
| 37.42 | 36.37 | 34.87 | 32.12 | 28.72 | 38.26 | 38.50 | 36.48 | 31.78 | 27.92 |
| 36.18 | 35.88 | 36.14 | 31.89 | 28.55 | 37.68 | N/A | 35.31 | 31.15 | 28.00 |
| 37.18 | 37.27 | 35.52 | 31.59 | 28.61 | 36.31 | N/A | 36.79 | 31.18 | 28.04 |
| 36.35 | 36.48 | 36.47 | 31.73 | 28.79 | N/A | 38.76 | 36.54 | 31.01 | 27.85 |
| 36.11 | 37.41 | 35.88 | 31.48 | 28.65 | 37.52 | 38.61 | 37.90 | 31.33 | 28.24 |
| 37.03 | 36.24 | 34.79 | 31.79 | 28.77 | 36.14 | 39.38 | 36.11 | 30.72 | 27.17 |
| 36.31 | N/A | 36.51 | 31.53 | 28.16 | N/A | 37.98 | N/A | 30.58 | 26.94 |
| 36.36 | N/A | 35.25 | 31.36 | 28.38 | N/A | 38.21 | 35.20 | N/A | 27.03 |
| 35.92 | 37.70 | 35.29 | N/A | 28.20 | 36.73 | 37.42 | 34.82 | 30.48 | 27.19 |
| 36.13 | 38.13 | 37.26 | 31.43 | 28.25 | N/A | 35.90 | 35.27 | 30.84 | 27.57 |
| UCDC-N3 | | | | | HKU-N | | | | |
| N/A | N/A | 38.19 | 32.37 | 28.83 | N/A | N/A | 39.87 | 34.06 | 30.28 |
| N/A | N/A | 36.67 | 32.07 | 28.87 | N/A | N/A | 38.33 | 33.67 | 30.20 |
| N/A | N/A | 36.90 | 32.07 | 28.92 | N/A | N/A | N/A | 33.45 | 30.18 |
| N/A | N/A | 37.22 | 31.91 | 28.92 | N/A | N/A | 38.95 | 33.50 | 30.26 |
| N/A | 39.91 | 38.19 | 32.27 | 29.07 | N/A | N/A | 38.51 | 33.58 | 30.34 |
| N/A | N/A | 36.14 | 32.05 | 28.65 | N/A | N/A | 38.18 | 33.41 | 30.44 |
| N/A | N/A | 35.60 | 32.06 | 28.50 | N/A | N/A | 38.60 | 33.54 | 30.31 |
| N/A | N/A | 36.74 | 32.21 | 28.64 | N/A | N/A | 38.02 | 33.52 | 30.30 |
| N/A | N/A | 36.55 | 31.95 | 28.82 | N/A | N/A | 39.27 | 33.41 | 30.39 |
| N/A | N/A | 37.16 | 32.10 | 29.14 | N/A | N/A | 37.94 | 33.19 | 30.25 |
| CCDC-N | | | | | Charité-E | | | | |
| 37.15 | 38.18 | 35.90 | 32.97 | 29.50 | N/A | 39.67 | 36.03 | 32.67 | 29.36 |
| 37.93 | 37.30 | 36.50 | 32.71 | 29.54 | N/A | N/A | 36.34 | 32.25 | 29.04 |
| 37.62 | 37.76 | 35.63 | 32.58 | 29.46 | N/A | N/A | 35.98 | 32.49 | 29.12 |
| 37.06 | 36.49 | 36.75 | 33.01 | 29.52 | N/A | N/A | 37.45 | 32.19 | 28.98 |
| 36.26 | 35.94 | 37.81 | 32.51 | 29.51 | N/A | 39.57 | 36.23 | 32.03 | 28.92 |
| N/A | 36.30 | 36.17 | 32.77 | 29.62 | N/A | N/A | 34.69 | 31.85 | 28.82 |
| N/A | 37.27 | 35.80 | 32.14 | 29.07 | N/A | N/A | 33.61 | 30.52 | 28.94 |
| N/A | 36.92 | 36.10 | 32.23 | 29.04 | N/A | N/A | 33.53 | 30.28 | 27.23 |
| N/A | N/A | 36.42 | 32.52 | 29.02 | N/A | N/A | 35.03 | 30.31 | 27.06 |
| N/A | N/A | 34.62 | 32.11 | 29.07 | N/A | N/A | 34.34 | 30.98 | 27.15 |
| HKU-ORF | | | | | CCDC-ORF | | | | |
| N/A | N/A | 37.66 | 32.98 | 29.13 | N/A | N/A | 36.09 | 32.63 | 28.81 |
| N/A | N/A | 37.67 | 32.47 | 28.88 | N/A | N/A | 35.60 | 32.48 | 29.00 |
| N/A | N/A | 38.70 | 32.32 | 28.67 | N/A | 39.44 | 37.35 | 32.19 | 29.04 |
| N/A | N/A | 36.63 | 32.21 | 28.65 | N/A | 38.76 | 37.02 | 32.74 | 28.94 |
| N/A | N/A | 39.10 | 32.62 | 29.15 | N/A | N/A | 34.06 | 32.51 | 29.03 |
| N/A | N/A | 37.47 | 32.53 | 29.45 | N/A | N/A | 36.96 | 30.93 | 27.77 |
| N/A | N/A | 37.00 | 32.37 | 29.39 | N/A | N/A | 35.09 | 31.55 | 27.96 |
| N/A | 39.53 | 37.20 | 32.57 | 29.51 | N/A | 35.47 | 34.51 | 31.70 | 28.03 |
| N/A | 39.49 | 37.38 | 32.41 | 29.61 | N/A | 37.46 | 34.95 | 31.37 | 27.91 |
| N/A | N/A | 38.82 | 32.51 | 29.65 | N/A | 35.70 | N/A | 31.09 | 27.73 |

**Table S2. Ct values of diluted samples with different primers/probes sets for RT-PCR.**

**Table S3. Concentrations of diluted samples with different primes/probes sets for ddPCR (copies/20 μl reaction).**

| UCDC-N1 | | | | | UCDC-N2 | | | | |
| --- | --- | --- | --- | --- | --- | --- | --- | --- | --- |
| MOCK | 10^-4^ | 10^-3^ | 10^-2^ | 10^-1^ | MOCK | 10^-4^ | 10^-3^ | 10^-2^ | 10^-1^ |
| 1.6 | 12 | 20 | 186 | 1660 | 1.6 | 1.6 | 20 | 142 | 1604 |
| 0 | 16 | 28 | 186 | 1840 | 0 | 3 | 20 | 142 | 1820 |
| 1.6 | 14 | 20 | 234 | 1780 | 0 | 0 | 11 | 166 | 1720 |
| 1.8 | 13 | 28 | 180 | 1820 | 1.4 | 1.8 | 24 | 178 | 1560 |
| 0 | 16 | 40 | 188 | 1740 | 0 | 0 | 18 | 174 | 1720 |
| 1.8 | 7 | 46 | 208 | 2320 | 0 | 3.8 | 11.2 | 178 | 1700 |
| 1.6 | 11 | 38 | 204 | 1900 | 1.8 | 4.2 | 12 | 142 | 1800 |
| 3 | 16 | 28 | 212 | 1860 | 1.8 | 4.2 | 12 | 186 | 1750 |
| 2.8 | 12 | 18 | 176 | 1940 | 1.6 | 6.2 | 14 | 142 | 1840 |
| 1.6 | 11 | 28 | 184 | 1760 | 0 | 4.2 | 22 | 160 | 1740 |
| UCDC-N3 | | | | | HKU-N | | | | |
| 0 | 0 | 16 | 136 | 1474 | 0 | 3.4 | 10.4 | 158 | 1620 |
| 0 | 0 | 22 | 128 | 1720 | 0 | 3.2 | 14 | 168 | 1716 |
| 0 | 1.8 | 20 | 146 | 1700 | 0 | 0 | 9.4 | 152 | 1580 |
| 0 | 1.8 | 14 | 164 | 1560 | 1.6 | 1.8 | 10.2 | 140 | 1660 |
| 0 | 2 | 10 | 196 | 1640 | 0 | 1.8 | 18 | 136 | 1800 |
| 0 | 0 | 10.6 | 170 | 1680 | 0 | 3.8 | 22 | 158 | 1700 |
| 0 | 0 | 11.2 | 148 | 1660 | 0 | 0 | 8.6 | 136 | 1660 |
| 0 | 3.4 | 14 | 156 | 1580 | 2.2 | 2 | 12.8 | 144 | 1642 |
| 0 | 0 | 10 | 122 | 1660 | 1.4 | 2.2 | 14 | 154 | 1780 |
| 1.6 | 3.4 | 11.2 | 178 | 1630 | 0 | 2 | 12.8 | 146 | 1642 |
| CCDC-N | | | | | Charité-E | | | | |
| 0 | 0 | 12.2 | 154 | 1660 | 0 | 1.4 | 14.2 | 104 | 1114 |
| 0 | 5.4 | 10.4 | 228 | 1900 | 0 | 0 | 5.6 | 98 | 1014 |
| 1.8 | 0 | 11.8 | 214 | 1980 | 0 | 0 | 11.8 | 124 | 1040 |
| 0 | 1.6 | 10.2 | 166 | 1900 | 0 | 0 | 10.8 | 148 | 910 |
| 0 | 5.4 | 12 | 182 | 1880 | 0 | 0 | 7 | 114 | 1080 |
| 0 | 0 | 15 | 190 | 1860 | 0 | 0 | 12.6 | 94 | 1022 |
| 0 | 0 | 14 | 244 | 1880 | 0 | 3 | 18 | 82 | 1116 |
| 0 | 4 | 12.6 | 204 | 1760 | 0 | 0 | 7 | 86 | 898 |
| 1.6 | 5.4 | 14 | 156 | 1820 | 0 | 1.6 | 7.4 | 88 | 832 |
| 0 | 1.4 | 15.2 | 162 | 1960 | 0 | 0 | 10.6 | 90 | 938 |
| HKU-ORF | | | | | CCDC-ORF | | | | |
| 0 | 0 | 4.4 | 102 | 802 | 0 | 1.6 | 7.2 | 102 | 976 |
| 0 | 1.6 | 4.6 | 74 | 572 | 0 | 0 | 7.8 | 98 | 1056 |
| 0 | 0 | 3.8 | 74 | 576 | 0 | 5.8 | 7.4 | 92 | 1068 |
| 0 | 0 | 3.8 | 56 | 642 | 0 | 0 | 6.8 | 86 | 1032 |
| 0 | 0 | 1.6 | 42 | 750 | 0 | 0 | 16 | 102 | 1004 |
| 0 | 0 | 1.6 | 42 | 782 | 0 | 0 | 10.4 | 94 | 970 |
| 0 | 0 | 8.2 | 48 | 710 | 0 | 3 | 8.6 | 100 | 966 |
| 0 | 0 | 7.6 | 62 | 664 | 0 | 3.4 | 15.8 | 120 | 1020 |
| 0 | 0 | 1.8 | 58 | 726 | 0 | 3.4 | 7.6 | 82 | 980 |
| 0 | 2 | 2.6 | 66 | 760 | 0 | 1.4 | 9.6 | 90 | 964 |

**Reference**

[1] Suo T, Liu X, Guo M, et al. ddPCR: a more sensitive and accurate tool for SARS-CoV-2 detection in low viral load specimens. medRxiv [Internet]. 2020;2020.02.29.20029439. Available from: http://medrxiv.org/content/early/2020/03/06/2020.02.29.20029439.abstract.

[2] National Institute For viral Disease Control and prevention of PRC. Specific primers and probes for detection 2019 novel coronavirus [Internet]. 2020 [cited 2020 Apr 10]. Available from: http://www.chinaivdc.cn/kyjz/202001/t20200121_211337.html.

[3] Centers for Disease Control and Prevention. 2019-Novel Coronavirus (2019-nCoV) Real-time RT-PCR Primer and Probe Information [Internet]. 2020 [cited 2020 Apr 10]. Available from: https://www.cdc.gov/coronavirus/2019-ncov/lab/rt-pcr-panel-primer-probes.html (2020).

[4] Corman VM, Landt O, Kaiser M, et al. Detection of 2019 novel coronavirus (2019-nCoV) by real-time RT-PCR. Euro Surveill. 2020;25:1–8.

[5] Chu DKW, Pan Y, Cheng SMS, et al. Molecular Diagnosis of a Novel Coronavirus (2019-nCoV) Causing an Outbreak of Pneumonia. Clin. Chem. 2020;66:549–555.
